# Co/Al Co-Substituted Layered Manganese-Based Oxide Cathode for Stable and High-Rate Potassium-Ion Batteries

**DOI:** 10.3390/ma17061277

**Published:** 2024-03-10

**Authors:** Junxian Li, Wenli Shu, Guangwan Zhang, Jiashen Meng, Chunhua Han, Xiujuan Wei, Xuanpeng Wang

**Affiliations:** 1 School of Materials Science and Engineering, Wuhan University of Technology, Wuhan 430070, China; 2 Sanya Science and Education Innovation Park, Wuhan University of Technology, Sanya 572000, China; 3 Hubei Longzhong Laboratory, Wuhan University of Technology (Xiangyang Demonstration Zone), Xiangyang 441000, China; 4 Institute for Sustainable Transformation, School of Chemical Engineering and Light Industry, Guangdong University of Technology, Guangzhou 510006, China; 5 Department of Physical Science & Technology, School of Science, Wuhan University of Technology, Wuhan 430070, China

**Keywords:** potassium-ion batteries, Mn-based layered oxide cathodes, synergy effect, Jahn-Teller effect, potassium storage mechanism

## Abstract

Manganese-based layered oxides are promising cathode materials for potassium-ion batteries (PIBs) due to their low cost and high theoretical energy density. However, the Jahn-Teller effect of Mn^3+^ and sluggish diffusion kinetics lead to rapid electrode deterioration and a poor rate performance, greatly limiting their practical application. Here, we report a Co/Al co-substitution strategy to construct a P3-type K_0.45_Mn_0.7_Co_0.2_Al_0.1_O_2_ cathode material, where Co^3+^ and Al^3+^ ions occupy Mn^3+^ sites. This effectively suppresses the Jahn-Teller distortion and alleviates the severe phase transition during K^+^ intercalation/de-intercalation processes. In addition, the Co element contributes to K^+^ diffusion, while Al stabilizes the layer structure through strong Al-O bonds. As a result, the K_0.45_Mn_0.7_Co_0.2_Al_0.1_O_2_ cathode exhibits high capacities of 111 mAh g^−1^ and 81 mAh g^−1^ at 0.05 A g^−1^ and 1 A g^−1^, respectively. It also demonstrates a capacity retention of 71.6% after 500 cycles at 1 A g^−1^. Compared to the pristine K_0.45_MnO_2_, the K_0.45_Mn_0.7_Co_0.2_Al_0.1_O_2_ significantly alleviates severe phase transition, providing a more stable and effective pathway for K^+^ transport, as investigated by in situ X-ray diffraction. The synergistic effect of Co/Al co-substitution significantly enhances the structural stability and electrochemical performance, contributing to the development of new Mn-based cathode materials for PIBs.

## 1. Introduction

In the current era of rapidly depleting fossil fuels and the growing demand for renewable energy, lithium-ion batteries (LIBs) have been widely used in various electronic devices due to their high energy density and long cycle life [1,2,3]. However, the huge consumption, rapidly increasing price and uneven distribution of lithium resources pose significant challenges to the sustainable development of LIBs. Consequently, there is a pressing need to identify suitable alternatives that are cost-effective and suitable for large-scale energy storage. In recent years, potassium-ion batteries (PIBs) have become highly promising candidates because of abundant resources and low cost [4,5,6,7]. PIBs have a similar operating mechanism to LIBs. And the low redox potential of K^+^/K (−2.93 V vs. SHE, second only to Li^+^/Li) gives it a high theoretical energy density. In addition, K^+^ exhibits fast diffusion kinetics in organic electrolytes because of its weak Lewis acidity [8,9]. However, the inherent property of the large K^+^ ionic radius (1.38 Å) greatly limits K^+^ transport in the electrode material and the electrode material undergoes severe volume changes during electrochemical cycling, leading to rapid capacity loss [10,11]. Therefore, the development of suitable electrode materials plays a crucial role in advancing PIB technology.

Currently, the main cathode materials being investigated for PIBs are Prussian blue analogues [12,13], polyanionic compounds [14,15] and layered transition metal oxides [16,17,18]. Among these, layered transition metal oxides are one of the most promising cathode materials thanks to the ease of synthesis and high theoretical capacity. Specifically, Mn-based layered oxides have garnered significant attention in PIBs owing to their low cost and environmental friendliness [19]. However, the Jahn-Teller distortion of Mn^3+^ leads to an unbalanced lengthening of the Mn-O bond and reduces the symmetry of the molecular structure, thus exacerbating the overall structural instability [20,21]. In addition, slow K^+^ transport, limited K^+^ storage sites and severe phase transitions during cycling cause their poor performance in practical applications [22]. Kim et al. [23] reported layered P3-K_0.5_MnO_2_ with a complex phase transition during cycling, leading to rapid capacity loss and retaining only 70% of the initial discharge capacity after 50 cycles at 20 mA g^−1^. Due to the poor diffusion of K^+^, this electrode shows a low specific capacity of only 38 mAh g^−1^ at 300 mA g^−1^, indicating a poor rate performance. Studies have shown that metal-ion substitution is a powerful measure to improve the above defects in K_x_MnO_2_. For example, Ni [24], Co [25], Fe [26], Mg [27] and Ni-Ti [28] substitutions have demonstrated the ability to improve their structural stability and electrochemical properties. Zhang et al. [29] reported a K_0.3_Mn_0.95_Co_0.05_O_2_ cathode. The Co doping suppressed Jahn-Teller distortion, allowing for more isotropic migration pathways for K^+^ in the interlayer. This enhanced the ionic diffusion and, consequently, the rate capability. Zhong et al. [30] found that Co-Fe co-substitution could expand the interlayer distance and effectively suppressed the interlayer-gliding to achieve highly reversible phase evolution, thus improving the rate performance and cycle life of K_0.5_MnO_2_.

Herein, we have modified K_0.45_MnO_2_ (KMO) with two selected metal elements (Co and Al), and have innovatively synthesized the co-substituted P3-K_0.45_Mn_0.7_Co_0.2_Al_0.1_O_2_ (KMCAO). Through the careful consideration of chemical bonding, Al^3+^ partially replaces the Mn^3+^ sites, forming stronger Al-O bonds compared to the Mn-O bonds. This substitution serves to stabilize the layer structure [31]. Additionally, the inclusion of Co^3+^ ions facilitated electrochemical reactions, providing active sites for K^+^ storage. Moreover, the Co^3+^/Al^3+^ co-substitution of Mn^3+^ sites effectively inhibited Jahn-Teller distortion, mitigating severe crystal structural transformations. Through in situ XRD, spherical aberration scanning transmission electron microscopy and first-principles simulation calculations, the KMCAO electrode exhibits a highly reversible phase structure transition during the cycling process. Meanwhile, Co/Al co-substitution widens the K layer spacing of the material and lowers the barrier energy for K^+^ migration. Compared to the pristine KMO, the KMCAO cathode exhibits superior cycling stability and rate performance. The excellent electrochemical performance of the KMCAO || soft carbon full cell also demonstrates its potential for practical applications. These findings highlight the effectiveness of our modification strategy and provide new insights into the development of PIB cathode materials.

## 2. Materials and Methods

All reactants are analytical grade. P3-type K_0.45_Mn_0.7_Co_0.2_Al_0.1_O_2_ and K_0.45_MnO_2_ samples are synthesized using a sol–gel method. Firstly, add 2 g of polyvinylpyrrolidone (PVP K90, Mw = 1,300,000) to 20 mL of deionized water and stir continuously to form a solution. KNO_3_, Mn(CH_3_COO)_2_·4H_2_O, Co(CH_3_COO)_2_·4H_2_O and Al(NO_3_)_3_·9H_2_O are dissolved into the solution by stoichiometric amounts. The mixed solution is dried at 80 °C for 15 h and then pre-sinters at 350 °C in air for 3 h to obtain a black solid. Finally, the black solid powder is calcined at 800 °C in air for 12 h to obtain K_0.45_Mn_0.7_Co_0.2_Al_0.1_O_2_. After slowly cooling down to 150 °C, the products are transferred promptly and stored in an Ar-filled glove box. The synthesis procedure of K_0.45_MnO_2_ is the same as that of K_0.45_Mn_0.7_Co_0.2_Al_0.1_O_2_, except that Co(CH_3_COO)_2_·4H_2_O and Al(NO_3_)_3_·9H_2_O are not added to the mixed solution.

The soft carbon is synthesized from 3,4,9,10-perylenetetracarboxylic dianhydride (PTCDA) after pyrolysis at 900 °C for 10 h under a flowing Ar atmosphere [32].

## 3. Results and Discussion

### 3.1. Structure and Morphology

**Figure 1:**
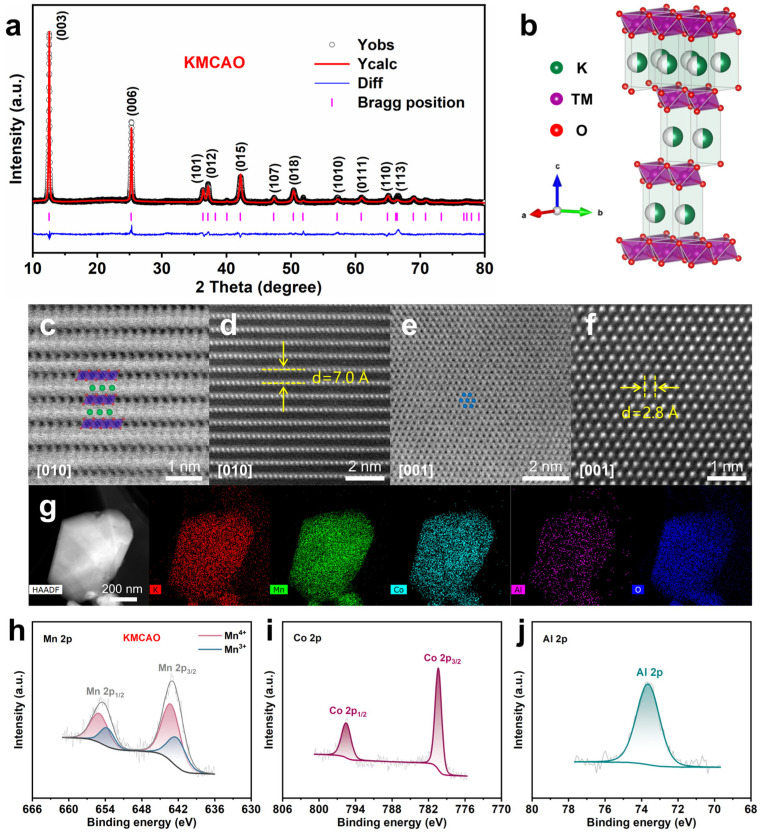
Structure characterizations of the KMCAO and KMO. (**a**) XRD Rietveld refinement of KMCAO. (**b**) P3 type structure schematic. (**c**) ABF-STEM and (**d**) HAADF-STEM images of KMCAO along the [010] zone axis. (**e**) ABF-STEM and (**f**) HAADF-STEM images of KMCAO along the [001] zone axis. (**g**) HAADF-STEM image of KMCAO and the corresponding EDS mappings for K, Mn, Co, Al and O elements. The XPS spectra of (**h**) Mn 2p, (**i**) Co 2p and (**j**) Al 2p of KMCAO.

The ICP test results of KMCAO and KMO are shown in Table S1. The chemical composition of the elements is consistent with the nominal composition. The electrical conductivities of KMCAO and KMO are measured using the four-probe method. The conductivity values obtained are 9.73 × 10^−6^ and 6.21 × 10^−6^ S cm^−1^, respectively (Table S2). Higher conductivity will favor the diffusion of ions, thus improving electrochemical performance. Figure 1a and Figure S1a show the Rietveld refinement XRD patterns of KMCAO and KMO. These patterns indicate that both samples exhibit a P3-type layered structure and belong to the *R3m* space group. Figure 1b shows the crystal structure of the P3 phase, where O ion layers are arranged in parallel in ABBCCA order, TM (Mn, Co, Al) ions occupy octahedral sites, and K ions occupy prismatic sites. The Rietveld refinement XRD reports of KMCAO and KMO are shown in Tables S3 and S4. The decreasing value of *a* (KMCAO: *a* = 2.8681 Å, KMO: *a* = 2.8745 Å) indicates that Co/Al co-substitution shrinks the TM layers, which helps stabilize the TM layers. The analysis of the cell parameters along the c-axis shows an enlargement of the K layers’ spacing, as evidenced by the increase in *c* value (KMCAO: *c* = 21.1069 Å, KMO: *c* = 20.8983 Å). This expansion is supported by the observed shift of the diffraction peak of the KMCAO (003) plane to a smaller angle in the XRD pattern (Figure S1b). The expansion of the interlayer space facilitates the diffusion of K ions and reduces the electrostatic repulsion between O ions in the neighboring TM layers. These effects contribute to the mitigation of phase transitions and interlayer sliding [25].

A spherical-aberration-corrected transmission electron microscope (AC-TEM) is used to obtain detailed atomic-scale crystal structure information of KMCAO through annular bright-field (ABF) and high-angle annular dark-field (HAADF) imaging. In the ABF-STEM images, the O and K layers are represented by bright grey dotted contrasts, while the TM layer appears as dark dotted contrasts. Conversely, the HAADF-STEM images display a reversal of contrast. In the ABF-STEM images, the arrangement of the K and TM layers in an alternating manner, as well as the stacking of the O layer according to the ABBCCA sequence along the [010] zone axis, can be observed (Figure 1c). This is a typical P3-phase structure. The HAADF-STEM image confirms that the distance between the neighboring layers is about 0.7 nm (Figure 1d), which corresponds to the Rietveld refinement XRD results (*c*/3). In addition, the ABF-STEM image reveals a hexagonal symmetry in the arrangement of the TM atoms along the [001] zone axis (Figure 1e). In the HAADF-STEM image, the measured distance between adjacent TM atoms is about 0.28 nm, which is consistent with the cell parameter *a* in the Rietveld refinement XRD results (Figure 1f). The detailed crystal structure of KMO can also be observed in the ABF-STEM and HAADF-STEM images (Figure S2). The scanning electron microscopy (SEM) and transmission electron microscopy (TEM) images (Figure S3a,b,d,e) reveal that both the KMCAO and KMO samples exhibit irregular polygonal shapes in terms of their particle morphology. The average particle diameter is approximately 1 μm. The high-resolution TEM (HRTEM) images show clear lattice stripes, indicating their high crystallinity. The lattice spacing is measured to be approximately 0.24 nm, corresponding to the (012) plane of the P3-type layered structure (Figure S3c,f). And both inset Selected Area Electron Diffraction (SAED) patterns show the hexagonal structure. Further, the energy-dispersive spectroscopy (EDS) mapping images demonstrate a uniform distribution of K, Mn, (Co, Al) and O elements throughout all the particles in the samples (Figure 1g and Figure S3g).

X-ray photoelectron spectroscopy (XPS) is used to characterize the surface chemical compositions and valences of the corresponding elements in KMCAO and KMO. The full spectrum clearly shows signals of K, Mn, Co, Al and O elements, confirming the successful co-substitution of Co and Al elements (Figure S4a). In the Co 2p spectrum of KMCAO, a double peak feature is observed at 780.1 eV and 795.2 eV, corresponding to Co 2p_3/2_ and Co 2p_1/2_, respectively, indicating the trivalent state of Co ions in the sample (Figure 1i) [33]. The Al 2p spectrum of the KMCAO exhibits a characteristic peak at 73.3 eV, corresponding to Al^3+^ (Figure 1j) [34]. The Mn 2p spectrum exhibits two main peaks that can be deconvoluted into four characteristic peaks (641.9 eV and 642.9 eV, 653.4 eV and 654.7 eV), which are attributed to Mn 2p_3/2_ and Mn 2p_1/2_, respectively. This indicates the presence of both Mn^3+^ and Mn^4+^ in the sample (Figure 1h and Figure S4b) [35]. It is worth noting that the area of the Mn^4+^ characteristic peaks is significantly larger in KMCAO compared to KMO, which effectively raises the average valence of Mn. The X-ray absorption near edge structure (XANES) tests are performed to further investigate the average oxidation state of Mn in KMCAO and KMO (Figure S4c). The comparison of the Mn_2_O_3_ and MnO_2_ reference spectra shows that the average oxidation state of Mn is an intermediate value between +3 and +4. The photon energy of the Mn K-edge of KMCAO shifts to a higher energy, indicating an increased oxidation state of Mn after Co/Al co-substitution. This finding is also consistent with the results obtained from theoretical chemical component valence calculations (Table S5). This co-substitution strategy could potentially mitigate the structural degradation caused by the Jahn-Teller effect of Mn^3+^, thereby enhancing the overall structural stability of the electrode materials.

### 3.2. Electrochemical Performance

**Figure 2:**
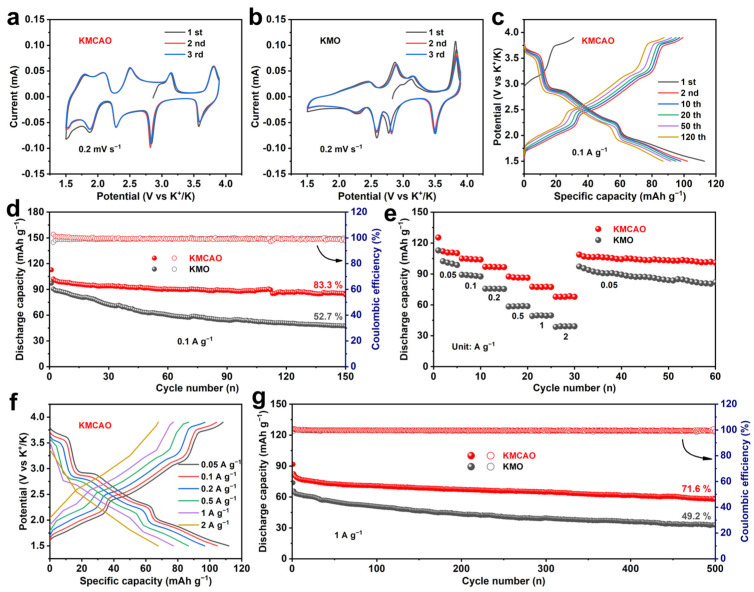
Electrochemical performance of KMCAO and KMO in 1.5~3.9 V (vs. K^+^/K). CV curves of the first three cycles at 0.2 mV s^−1^ of (**a**) KMCAO and (**b**) KMO. (**c**) Charge/discharge curves of KMCAO at 0.1 A g^−1^. (**d**) Cycling performance at 0.1 A g^−1^. (**e**) Rate performance at 0.05, 0.1, 0.2, 0.5, 1 and 2 A g^−1^. (**f**) The corresponding charge/discharge curves of KMCAO at different rates. (**g**) Long-term cycling performance at 1 A g^−1^.

The K storage performance of the prepared KMCAO and KMO cathodes was evaluated using cyclic voltammetry (CV) and constant current charge/discharge tests within the voltage range of 1.5~3.9 V. Figure 2a,b show the typical CV curves for KMCAO and KMO cathodes, respectively, at a scan rate of 0.2 mV s^−1^. KMCAO shows five pairs of redox peaks. It is generally believed that the pair of redox peaks at 1.78/1.50 V may be related to the transformation of K^+^/vacancy order/disorder due to the mixing of transition metal ions [36]. And the two pairs of redox peaks at 2.08/1.87 V and 2.50/2.28 V are attributed to the Mn^3+^/Mn^4+^ redox pair, while the other two pairs of redox peaks at 2.84/3.14 V and 3.58/3.81 V are associated with Mn^3+^/Mn^4+^ and Co^3+^/Co^4+^ contributions [37,38]. The redox peaks in KMO are all attributed to Mn^3+^/Mn^4+^ [18]. Compared to the pristine KMO, the Co/Al co-substitution significantly reduces the potential interval between the oxidation and reduction peaks, and the reduction in polarization will be beneficial for its practical application. Table S6 shows the calculation of voltage polarization. In addition, the better overlap of the CV curves indicates that KMCAO has remarkable reversibility during the electrochemical reaction process. The participation of active Co elements in the electrochemical reaction contributes to additional capacity, while the electrochemically inactive Al element stabilizes the layer structure through robust Al-O bonding [39]. Figure 2c and Figure S5 show the charge/discharge curves of KMCAO and KMO cathodes for different cycles at a rate of 0.1 A g^−1^. The voltage plateaus correspond to the redox peaks. Co/Al co-substitution in KMO can effectively smooth the charge–discharge profiles and increase the reversible capacity. At 0.1 A g^−1^, KMCAO exhibits a high reversible discharge specific capacity of 102 mAh g^−1^, with a capacity retention of 83.3% after 150 cycles. In contrast, the KMO cathode shows a rapid capacity decrease from 91 mAh g^−1^ to 48 mAh g^−1^ after 150 cycles, corresponding to a capacity retention of only 52.7% (Figure 2d). The SEM and TEM images after cycling for KMCAO and KMO are presented in Figure S6. The particle morphology of both KMCAO and KMO are preserved well after cycling. In comparison, KMCAO has a better integrity of particle morphology. In addition, the KMCAO cathode has an excellent rate performance with average discharge specific capacities of 111, 104, 96, 87, 77 and 67 mAh g^−1^ at 0.05, 0.1, 0.2, 0.5, 1 and 2 A g^−1^, respectively. When the current density is reset to 0.05 A g^−1^, a discharge specific capacity of 108 mAh g^−1^ can be obtained, which is close to the initial value (Figure 2e). The corresponding charge/discharge curves demonstrate the rapid K^+^ storage property and low polarization characteristics of the KMCAO cathode (Figure 2f and Figure S7). The KMCAO cathode also exhibits a long cycle lifespan. After 500 cycles at 1 A g^−1^, the discharge specific capacity is 58 mAh g^−1^, corresponding to a capacity retention of 71.6%. In comparison, the KMO cathode retains only 49.2% of its capacity (Figure 2g). This improved cycling performance can be attributed to the successful regulation of the Mn average valence through Co/Al co-substitution, which suppresses the Jahn-Teller effect. Notably, the KMCAO cathode demonstrates competitive K^+^ storage performance compared to previously reported layered oxide cathodes for PIBs (Table S7). The electrochemical performance of the high mass loadings of KMCAO is investigated (Figure S8). It also performs well. After 100 cycles at 0.1 A g^−1^, the KMCAO with a high mass loading of 7.11 mg cm^−2^ maintains a high reversible capacity of 64 mAh g^−1^ and capacity retention of 77.1%. Also, the KMCAO demonstrates exceptional rate performance even with a high mass loading of 6.34 mg cm^−2^. The average discharge capacities are 88, 81, 74, 65 and 54 mAh g^−1^ at 0.1, 0.2, 0.5, 1 and 2 A g^−1^, respectively. Further, galvanostatic intermittent titration technique (GITT) and electrochemical impedance spectroscopy (EIS) tests are performed to investigate the K^+^ diffusion dynamics of the KMCAO and KMO cathodes. Based on the GITT test results (Figure S9a,b), the K^+^ diffusion coefficient of KMCAO is calculated to be 10^−9^–10^−11^ cm^2^ s^−1^ during the first discharge cycle, which is generally higher than that of the pristine KMO cathode (Figure S9c). The EIS fitting results show that the KMCAO cathode has a lower charge transfer resistance and faster K^+^ diffusion dynamics compared to the KMO cathode (Figure S10 and Table S8).

### 3.3. Potassium Storage Mechanism

**Figure 3:**
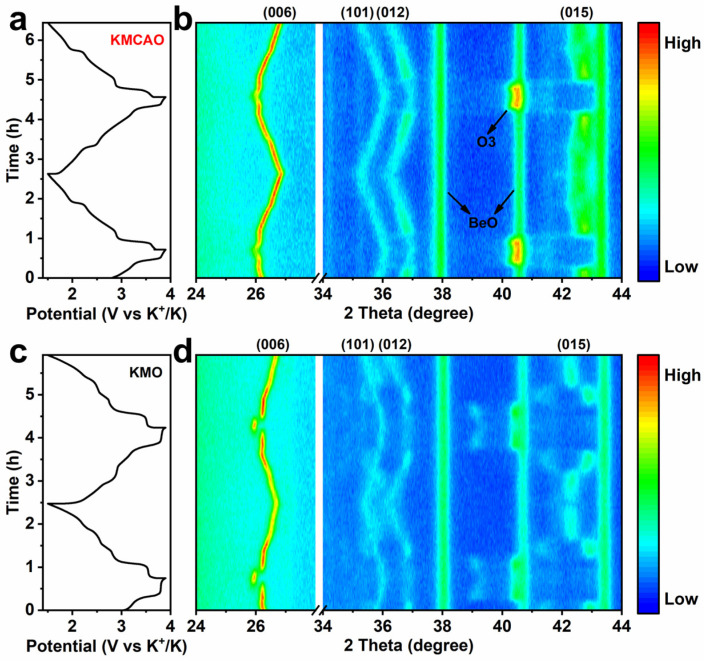
In situ XRD characterization of (**a**,**b**) KMCAO and (**c**,**d**) KMO at 50 mA g^−1^ in the range of 1.5~3.9 V.

In order to investigate the detailed crystal structure evolution during the K^+^ intercalation/de-intercalation process, in situ XRD experiments are conducted to monitor the charging/discharging process of both the KMCAO and KMO cathodes (Figure 3). During the charging process, the (006) plane of KMCAO gradually shifts to a lower angle, while the (101), (012) and (015) planes shift to higher angles. These observations indicate the detachment of K^+^. K^+^ acts as an electrostatic shield between the O layers. With K^+^ extraction, the shielding effect weakens and the electrostatic repulsion between O ions dominates. This leads to an expansion of the *c*-axis and a contraction of the *a*-*b* plane [40]. At higher voltages, the (015) peak disappears and a new diffraction peak appears at about 40.5°, corresponding to the (104) plane of the O3 phase. During discharge, the (015) peak reappears as the voltage decreases. At this stage, the electrostatic repulsion between the O layers decreases with the re-insertion of K^+^. The (006) plane shifts back to a higher angle, and the (101), (012) and (015) planes shift to lower angles, restoring the initial P3 phase state. These shift behaviors of typical planes are the same in the first two charge/discharge cycles, showing the highly reversible K^+^ intercalation/de-intercalation process (Figure 3a,b). In the case of KMO, the phase transition between P3 and O3 also occurs in the high-potential zone. However, a notable difference arises as the (006) plane experiences a sudden jump during the phase transition, corresponding to an abrupt change in the lattice parameter *c* value, which shows a drastic lattice distortion in KMO. On the other hand, the *c* value of KMCAO changes smoothly throughout the charging/discharging process (Figure S11). The (104) plane also exhibits a jump during the process (Figure 3c,d). In addition to the phase transition in the high-potential zone, the intensity of the KMO (006) plane diminishes in the low-potential zone (discharging to ~2.5 V and charging to ~2.8 V), indicating a weakening of the P3 phase feature. Furthermore, indistinguishable diffraction peaks appear near the (101) and (012) planes, while the (015) peak shows jumping displacements and disconnections. Therefore, we suggest that these complex and severe phase transitions may accelerate the further degradation of the lattice structure of the KMO cathode during charging/discharging, leading to the poor cycle stability observed during K^+^ intercalation/de-intercalation [41].

### 3.4. First-Principles Calculations

**Figure 4:**
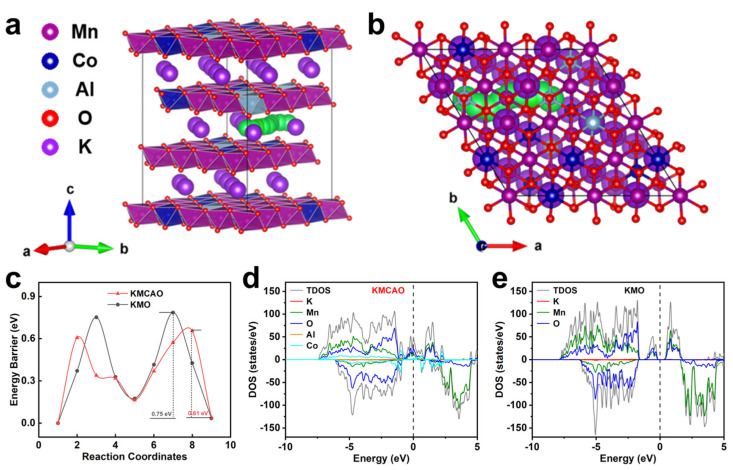
First-principles calculations. Schematic of the KMCAO crystal structure showing the K^+^ migration pathways (indicated by the green balls) from (**a**) the side and (**b**) the top views. (**c**) The corresponding migration energy barriers in KMCAO and KMO. Density of states of (**d**) KMCAO and (**e**) KMO.

First-principles calculations are conducted to further investigate the reasons for the superior electrochemical performance of the KMCAO cathode. First, based on the crystal structures of KMCAO (Figure 4a,b) and KMO (Figure S12a,b), the transition state structures are constructed using the Climbing Image Nudged Elastic Band (CINEB) method with linear interpolation points [42], illustrating the path for K^+^ migration (the green balls in the figures). Subsequently, the migration energy barrier of K^+^ in the KMCAO lattice is calculated to be about 0.61 eV (Figure 4c). The lower migration energy barriers favor K^+^ intercalation and de-intercalation, thereby facilitating highly reversible redox reactions. In addition, the density of states (DOS) show that KMCAO exhibits a continuous distribution of the density of states near the Fermi energy level, with a significantly higher intensity of the density of states compared to KMO, which has a clear band gap near the Fermi energy level. This confirms the enhanced electrical conductivity of the KMCAO material. These results provide conclusive evidence for the excellent electrochemical performance of the KMCAO cathode. Overall, the Co/Al co-substitution offers more active channels for K^+^ diffusion, which helps alleviate the irreversible phase transition during the reaction, thereby ensuring the maintenance of the structural stability of the crystals.

### 3.5. Full Cell Demonstration

**Figure 5:**
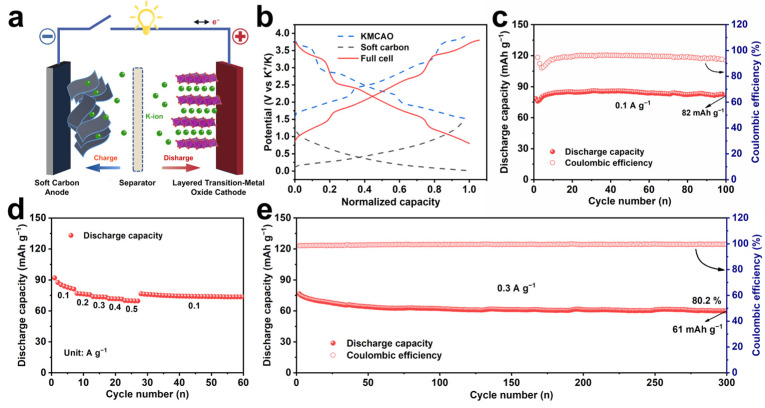
Electrochemical performance of potassium-ion full cell based on KMCAO/soft carbon in 0.8~3.8 V. (**a**) Schematic illustration of the cell configuration and operational mechanism of the full PIBs. (**b**) Normalized charge/discharge curves of the half and full PIBs. (**c**) Cycling performance at 0.1 A g^−1^. (**d**) Rate performance at 0.1, 0.2, 0.3, 0.4 and 0.5 A g^−1^. (**e**) Long-term cycling capability at 0.3 A g^−1^.

To assess the practical application potential of the KMCAO cathode, performance tests are conducted on a potassium-ion full cell, paired with a soft carbon anode (Figure 5). The structural and morphological characterizations of the soft carbon are shown in Figure S13. And Figure S14 shows its electrochemical performances. Before assembling the full cell, the soft carbon anode was pre-cycled at 0.01~1.5 V (vs. K^+^/K) to activate the material. The anode/cathode capacity ratio is adjusted to 1.2 to eliminate irreversibility. The configuration and operational mechanism of typical full PIBs are shown in Figure 5a. Figure 5b shows the normalized charge/discharge curves of KMCAO and soft carbon electrodes in half/full PIBs. The full cell maintains a high specific capacity of 82 mAh g^−1^ after 100 cycles at 0.1 A g^−1^ (Figure 5c). It also exhibits a good rate performance, with average discharge specific capacities of 85, 76, 73, 71 and 69 mAh g^−1^ at 0.1, 0.2, 0.3, 0.4 and 0.5 A g^−1^, respectively (Figure 5d). When tested at 0.3 A g^−1^, the initial discharge specific capacity of the full cell was 76 mAh g^−1^ and the initial coulombic efficiency was close to 99%. And the capacity remains at 80.2% after 300 cycles. (Figure 5e). These results highlight that KMCAO cathode has a great deal of potential for practical applications in PIBs.

## 4. Conclusions

In summary, the successful synthesis of a Co/Al co-substituted P3-type layered KMCAO cathode for PIBs has been achieved. The incorporation of Co^3+^/Al^3+^ into the Mn^3+^ sites effectively suppresses the Jahn-Teller distortion. Additionally, Co contributes to the electrochemical reaction, enhancing capacity, while Al forms stable bonds with oxygen, further stabilizing the layer structure. The KMCAO cathode exhibits a specific discharge capacity of 111 mAh g^−1^ at 0.05 A g^−1^. It also shows an excellent rate performance with a specific capacity of 81 mAh g^−1^ at 1 A g^−1^ and retains 71.6% of its capacity after 500 cycles. The Co/Al co-substitution also results in a wider spacing between the K layers, reduced K^+^ diffusion barriers and faster K^+^ diffusion dynamics compared to the pristine KMO cathode. Furthermore, the in situ XRD results show that the KMCAO cathode exhibits a milder phase transition during electrode cycling. The Co/Al co-substitution strategy employed in this work provides an effective approach for designing high-performance cathode materials in PIBs.

## Data Availability

Data are contained within the article and Supplementary Materials.

## Supplementary Materials

The following supporting information can be downloaded at: https://www.mdpi.com/article/10.3390/ma17061277/s1. Figure S1. (a) XRD Rietveld refinement of KMO. (b) Zoomed-in image of XRD patterns of KMCAO and KMO for (003) peaks. Figure S2. (a) ABF-STEM and (b) HAADF-STEM images of KMO along the [010] zone axis. (c) ABF-STEM and (d) HAADF-STEM images of KMO along the [001] zone axis. Figure S3. (a) SEM, (b) TEM and (c) HRTEM images of KMCAO. (d) SEM, (e) TEM and (f) HRTEM images of KMO (inset: SEAD pattern). (g) HAADF-STEM image of KMO and the corresponding EDS mappings for K, Mn and O elements. Figure S4. (a) The XPS spectra of KMCAO and KMO. (b) Mn 2p of KMO. (c) XANES spectra of Mn K-edge. Figure S5. Charge/discharge curves of KMO at 0.1 A g^−1^. Figure S6. SEM images after 150 cycles at 0.1 A g^−1^ for (a) KMCAO and (b) KMO. TEM images after 150 cycles at 0.1 A g^−1^ for (c) KMCAO and (d) KMO. Figure S7. The corresponding charge/discharge curves of KMO at different rates. Figure S8. Cycling and rate performances of KMCAO at the high mass loadings. (a) Charge/discharge curves at 0.1 A g^−1^ (b) Cycling performance with coulombic efficiencies measured at 0.1 A g^−1^. (c) Rate performance conducted at 0.05, 0.1, 0.2, 0.5, 1 and 2 A g^−1^. Figure S9. Potential response of (a) KMCAO and (b) KMO during GITT measurements. (c) The calculated diffusion coefficient of K^+^ for KMCAO and KMO. Figure S10. Nyquist plots and the equivalent circuit model. Figure S11. Lattice parameter *c* variation of (a) KMCAO and (b) KMO during the second charge/discharge process. Figure S12. Schematic crystal structure of KMO showing the K^+^ migration pathways (described by the green balls) from (a) the side and (b) the top views. Figure S13. Structural and morphological characterizations of soft carbon. (a) XRD pattern. (b) SEM image. (c) Raman spectrum. Figure S14. Electrochemical performances in the potential range of 0.01–1.5 V of soft carbon. (a) Charge/discharge curves at 0.1 A g^−1^. (b) Cycling performance at 0.1 A g^−1^. (c) Rate performance at 0.1, 0.2, 0.5, 1 and 2 A g^−1^. Table S1. ICP measurement results of KMCAO and KMO. Table S2. The resulting electrical conductivities of KMCAO and KMO measured by the four-point probe method. Table S3. Structural parameters and atomic position of KMCAO from Rietveld refinement. Table S4. Structural parameters and atomic position of KMO from Rietveld refinement. Table S5. Mn average valence calculation results of K_0.45_Mn_0.7_Co_0.2_Al_0.1_O_2_ and K_0.45_MnO_2._ Table S6. The voltage polarization calculation results of K_0.45_Mn_0.7_Co_0.2_Al_0.1_O_2_ and K_0.45_MnO_2._ Table S7. Electrochemical performance of K_0.45_Mn_0.7_Co_0.2_Al_0.1_O_2_ compared with other layered oxide cathodes in PIBs. Table S8. The obtained resistance values for K_0.45_MnO_2_ and K_0.45_Mn_0.7_Co_0.2_Al_0.1_O_2_ through fitting the EIS spectra along the equivalent circuit. Refs. [28,40,43,44,45,46,47,48,49,50,51] are cited in the Supplementary Materials.
