# Supplementary material for: Co/Al Co-Substituted Layered Manganese-Based Oxide Cathode for Stable and High-Rate Potassium-Ion Batteries
Source: Materials (Basel). 2024 Mar 10;17(6):1277. doi: 10.3390/ma17061277 (PMC10972413; doi:10.3390/ma17061277)
Supplement: Supplementary file 1 [file materials-17-01277-s001.zip › materials-2904390-supplementary.pdf]

Supporting information

Co/Al Co-Substituted Layered Manganese-Based Oxide
Cathode for Stable and High-Rate Potassium-Ion Batteries

Junxian Li [1,2], Wenli Shu [1,2], Guangwan Zhang [1,2], Jiashen Meng [1], Chunhua Han [1,2,3], Xiujuan Wei [4,*] and Xuanpeng Wang [1,2,3,5,*]

[1] School of Materials Science and Engineering, Wuhan University of Technology, Wuhan 430070, China
[2] Sanya Science and Education Innovation Park, Wuhan University of Technology, Sanya 572000, China
[3] Hubei Longzhong Laboratory, Wuhan University of Technology (Xiangyang Demonstration Zone), Xiangyang 441000, China
[4] Institute for Sustainable Transformation, School of Chemical Engineering and Light Industry, Guangdong University of Technology, Guangzhou 510006, China
[5] Department of Physical Science & Technology, School of Science, Wuhan University of Technology,
Wuhan 430070, China
* Correspondence: xjwei@gdut.edu.cn (X.W.); wxp122525691@whut.edu.cn (X.W.); Tel.: +86-135-4528-4506 (Xuanpeng Wang)

Characterization

XRD patterns were obtained on a D8 Discover X-ray diffractometer with a non-monochromated Cu Kα X-ray as the source (λ = 1.5406 Å). XRD Rietveld refinement was performed using TOPAS academic software. ICP measurement was recorded with a PerkinElmer Optima 4300DV spectrometer. SEM measurement was measured with a JEOL JSM-7100F with 20 kV acceleration voltage. TEM, HAADF-STEM and HRTEM images were collected with a JEM-2100F and a Thermo Fischer Titan G2 60-300 microscope. The elemental mapping was conducted with an EDX-GENESIS 60S spectrometer. The STEM characterization was

performed on a Thermo Fischer Titan Themis STEM with 300 kV acceleration voltage. The STEM images were obtained with an ABF detector and a HAADF detector, respectively. XPS test was performed with a VG MultiLab 2000 instrument. For the in situ XRD measurement, $K_{0.45}Mn_{0.7}Co_{0.2}Al_{0.1}O_2$ and $K_{0.45}MnO_2$ cathodes were charged to 3.9 V and then discharged to 1.5 V (vs. K^+/K) at 50 mA g^{-1}, with 2θ within range from 24° to 44°. The electrochemical tests were measured by using CR2016 coin cells.

Electrochemical measurements

The working electrode was prepared by casting a mixture of active material, acetylene black and polyvinylidene fluoride (PVDF) binder with a weight ratio of 70: 20: 10 onto Al foil, followed by drying at 70 ℃ in a glovebox for 10 h. The active mass loading of cathodes was about 1.2-2.0 mg cm^{-2} per electrode. The electrolyte was a solution of 0.8 M potassium hexafluorophosphate (KPF6) in ethylene carbonate (EC) and diethyl carbonate (DEC) (1:1 in volume). Potassium metal foil was used as the counter electrode and porous glass microfiber filter (Grade GF/D Whatman) was used as the separator. For the full cell, the anode was composed of 70 wt % soft carbon, 20 wt % acetylene black and 10 wt % PVDF binder and coated onto Cu foil. The area loading of anode materials was approximately 0.7-1.2 mg cm^{-2}. Pre-cycling of soft carbon materials:

10 complete charge/discharge cycles at 0.1 A g^{-1} in the range 0.01-1.5 V (vs. K^+/K) to activate the electrodes. The cycled electrodes replaced the original potassium metal sheets as the anode, and KMCAO as the cathode to assemble a full potassium-ion cell. The separator and electrolyte from the pre-cycling could continue to be used in the full cell. Galvanostatic discharge/charge tests were carried out using a LAND CT2001A multichannel testing system. EIS and CV measurements were conducted with an Auto lab PGSTAT 302N and CHI 600e electrochemical workstation. GITT curve was also measured using a LAND CT2001A multichannel testing system, which conducted at a pulse current of 10 mA g^{-1} for 5 min, followed with a relaxation for 30 min. The ionic diffusion coefficient was calculated through the following simplified equation:

$$D^{GITT} = \frac{4}{\pi\tau}\left(\frac{m_B V_M}{M_B S}\right)^2 \left(\frac{\Delta E_S}{\Delta E_\tau}\right)^2$$

where V_M, M_B, m_B and S represent the molar volume, the molecular weight, the mass and the active surface area of the electrode (the diameter of the electrode sheet is 10 mm, so the active surface area is 0.785 cm^2), respectively; ΔE_τ and ΔE_s are the change in the cell voltage during the current pulse and the steady-state voltage during the step at the plateau potential, respectively.

First principles calculations

All first-principles calculations were performed in the Vienna Ab initio Simulation Package (VASP) software using projection augmented wave (PAW) with periodic boundary conditions in the framework of density functional theory (DFT) [1, 2]. The exchange-correlation energy was calculated using the generalized gradient approximation (GGA) of the Perdew–Burke–Ernzerhof (PBE) formulation [3]. The Brillouin zone integration was performed with a 0.04 $Å^{-1}$ *k*-mesh Monkhorst–Pack sampling [4]. The self-consistent calculations applied a convergence energy threshold of 10^{-5} eV. The spin polarization method was adopted to describe the magnetic system. Both structural optimizations and electronic structure calculations were carried out by using the spin-dependent GGA plus Hubbard correction U method, and the effective U_{eff} parameter was 3.9 eV for Mn atom and 3.32 eV for Co. The Climbing Image Nudged Elastic Band (CI-NEB) method was used to investigate the minimum energy pathway [5].

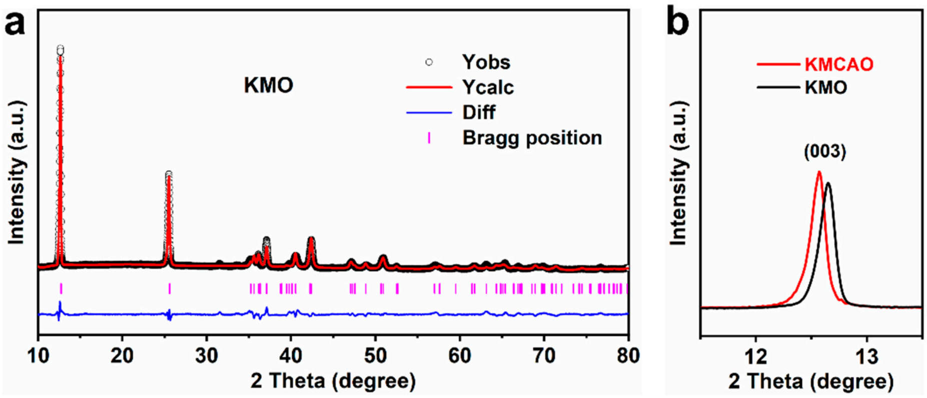

Figure S1. (a) XRD Rietveld refinement of KMO. (b) Zoomed-in image of XRD patterns of KMCAO and KMO for (003) peaks.

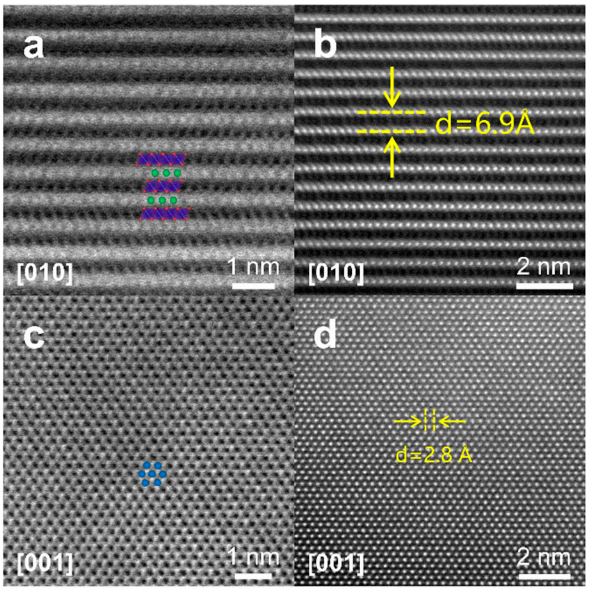

Figure S2. (a) ABF-STEM and (b) HAADF-STEM images of KMO along the [010] zone axis. (c) ABF-STEM and (d) HAADF-STEM images of KMO along the [001] zone axis.

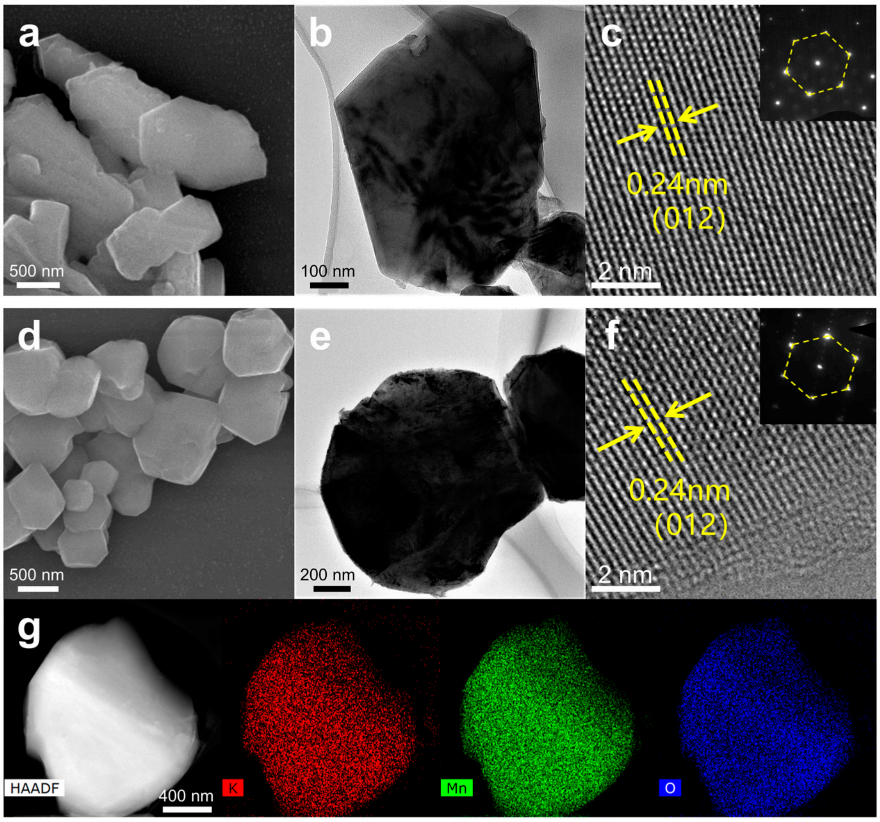

Figure S3. (a) SEM, (b) TEM and (c) HRTEM images of KMCAO. (d) SEM, (e) TEM and (f) HRTEM images of KMO (inset: SEAD pattern). (g) HAADF-STEM image of KMO and the corresponding EDS mappings for K, Mn and O elements.

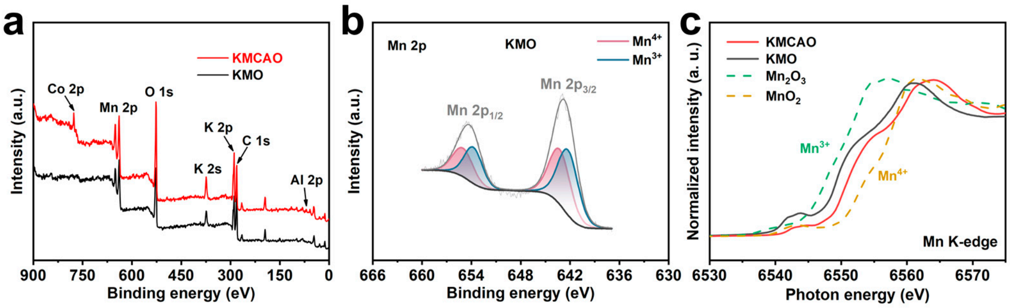

Figure S4. (a) The XPS spectra of KMCAO and KMO. (b) Mn 2p of KMO. (c) XANES spectra of Mn K-edge.

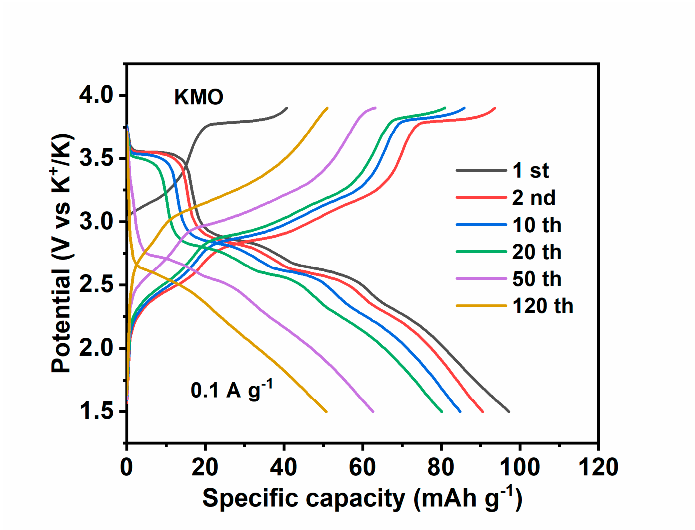

Figure S5. Charge/discharge curves of KMO at 0.1 A g^{-1}.

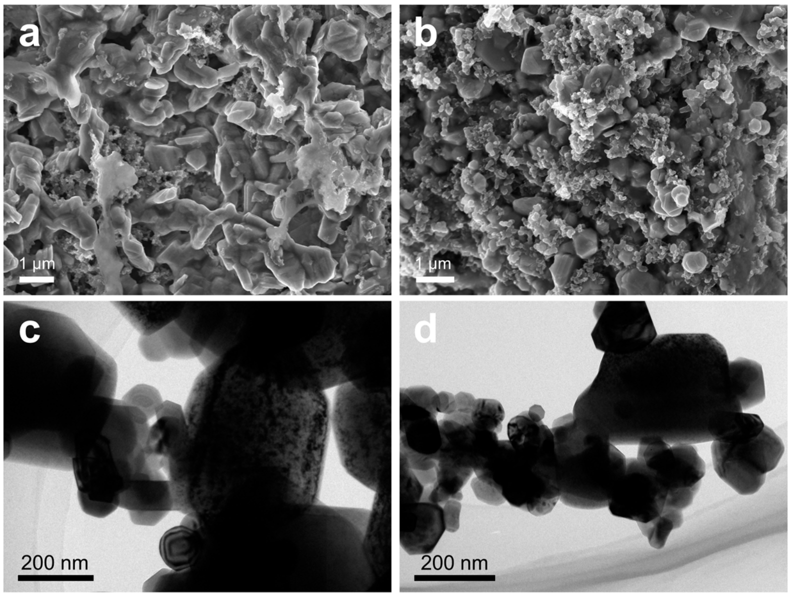

Figure S6. SEM images after 150 cycles at 0.1 A g^{-1} for (a) KMCAO and (b) KMO. TEM images after 150 cycles at 0.1 A g^{-1} for (c) KMCAO and (d) KMO.

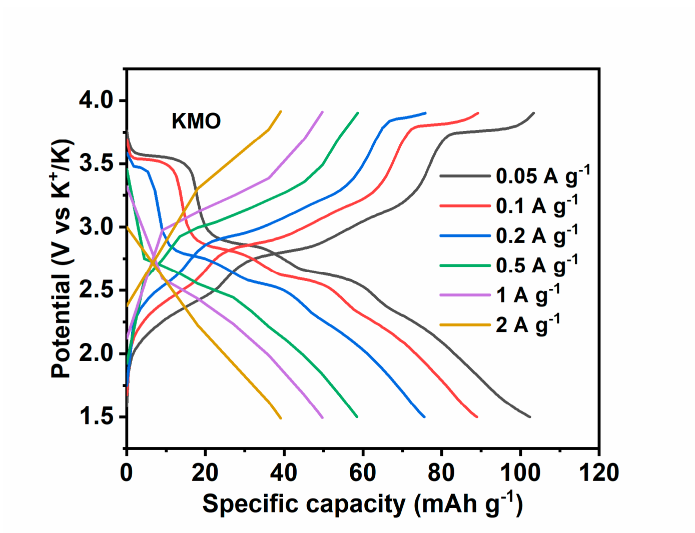

Figure S7. The corresponding charge/discharge curves of KMO at different rates.

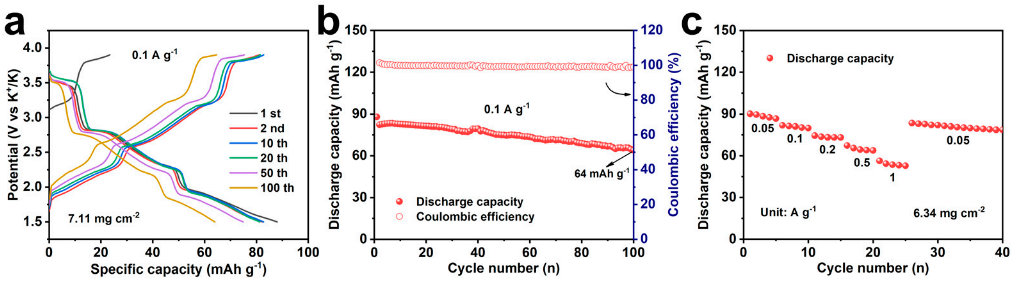

Figure S8. Cycling and rate performances of KMCAO at the high mass loadings. (a) Charge/discharge curves at 0.1 A g^{-1} (b) Cycling performance with coulombic efficiencies measured at 0.1 A g^{-1}. (c) Rate performance conducted at 0.05, 0.1, 0.2, 0.5, 1 and 2 A g^{-1}.

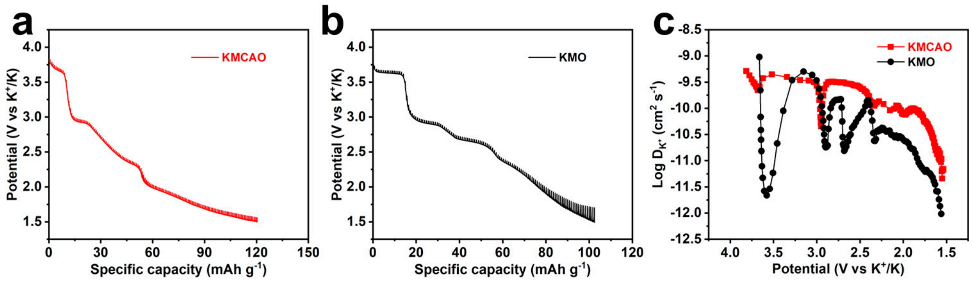

Figure S9. Potential response of (a) KMCAO and (b) KMO during GITT measurements. (c) The calculated diffusion coefficient of K^+ for KMCAO and KMO.

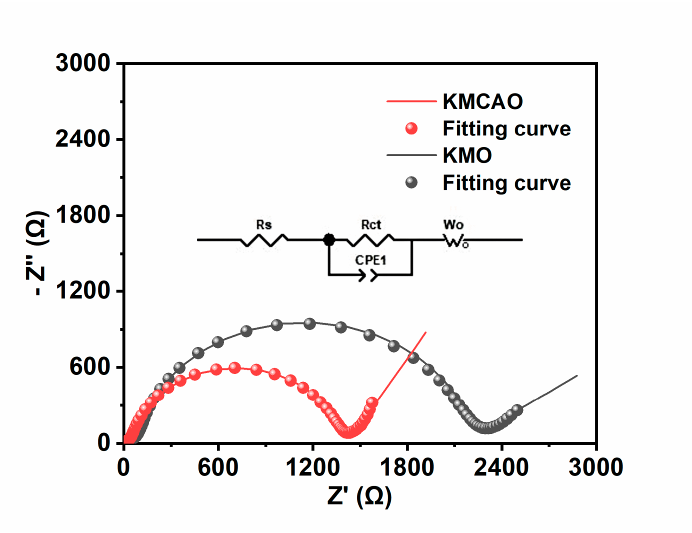

Figure S10. Nyquist plots and the equivalent circuit model.

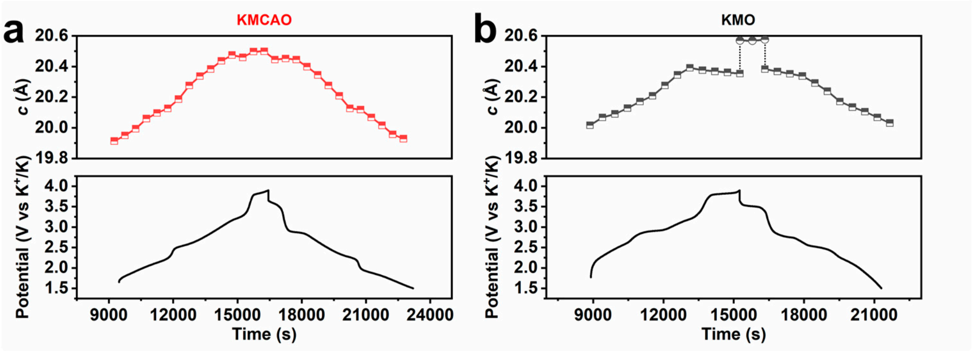

Figure S11. Lattice parameter *c* variation of (a) KMCAO and (b) KMO during the second charge/discharge process.

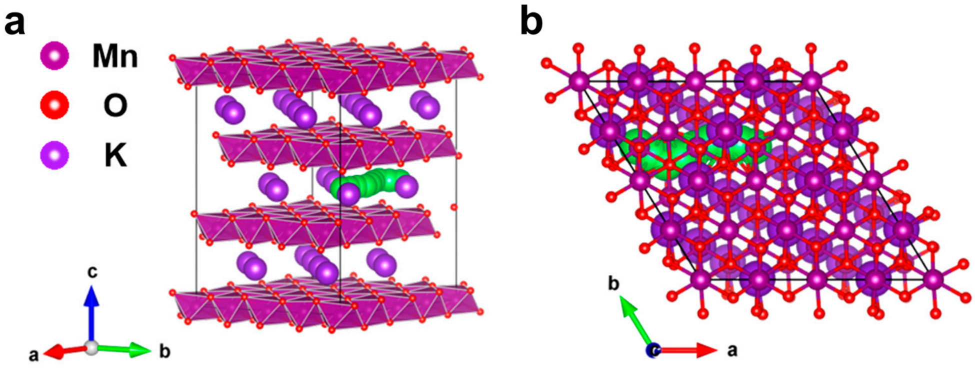

Figure S12. Schematic crystal structure of KMO showing the K^+ migration pathways (described by the green balls) from (a) the side and (b) the top views.

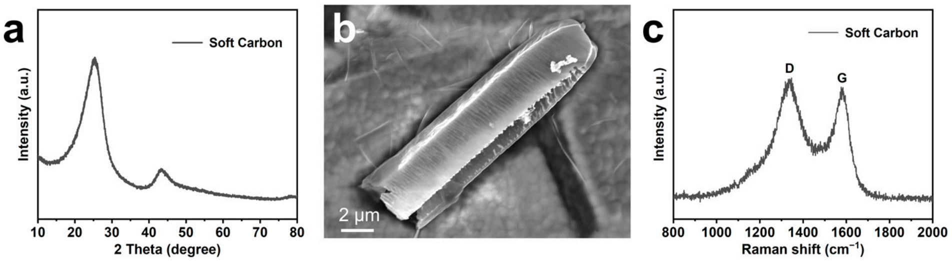

Figure S13. Structural and morphological characterizations of soft carbon. (a) XRD pattern. (b) SEM image. (c) Raman spectrum.

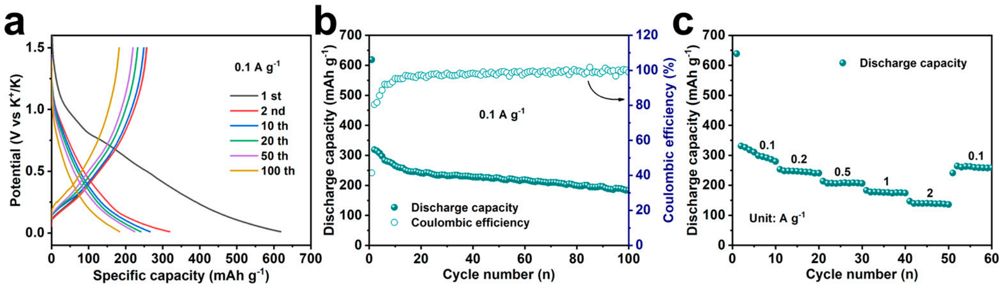

Figure S14. Electrochemical performances in the potential range of 0.01-1.5 V of soft carbon. (a) Charge/discharge curves at 0.1 A g^{-1}. (b) Cycling performance at 0.1 A g^{-1}. (c) Rate performance at 0.1, 0.2, 0.5, 1 and 2 A g^{-1}.

Table S1. ICP measurement results of KMCAO and KMO.

Theoretical chemical formula	K: Mn (: Co: Al)
$K_{0.45}Mn_{0.7}Co_{0.2}Al_{0.1}O_2$	0.458: 0.700: 0.206: 0.106
$K_{0.45}MnO_2$	0.454: 1.000

Table S2. The resulting electrical conductivities of KMCAO and KMO measured by the four-point probe method.

	σ (S cm^{-1})
$K_{0.45}Mn_{0.7}Co_{0.2}Al_{0.1}O_2$	9.73×10^{-6}
$K_{0.45}MnO_2$	6.21×10^{-6}

Table S3. Structural parameters and atomic position of KMCAO from Rietveld refinement.

Formula		$K_{0.45}Mn_{0.7}Co_{0.2}Al_{0.1}O_2$	
Crystal system		Hexagonal	
Space group		*R3m*	
Atom	*x*	*y*	*z*
K	0	0	0.841
Mn	0	0	0
Co	0	0	0
Al	0	0	0
O	0	0	0.381
O	0	0	0.612
a= *b*(Å)		2.8681	
c(Å)		21.1069	
Cell volume (Å^3)		150.362	
Crystal density (g cm^{-3})		3.691	
R_{wp} (%)		7.10	
R_p (%)		5.08	

Table S4. Structural parameters and atomic position of KMO from Rietveld refinement.

Formula		$K_{0.45}MnO_2$	
Crystal system		Hexagonal	
Space group		*R3m*	
Atom	*x*	*y*	*z*
K	0	0	0.850
Mn	0	0	0
O	0	0	0.380
O	0	0	0.611
a= *b*(Å)		2.8745	
c(Å)		20.8983	
Cell volume (Å^3)		149.547	
Crystal density (g cm^{-3})		3.718	
R_{wp} (%)		8.49	
R_p (%)		6.16	

Table S5. Mn average valence calculation results of $K_{0.45}Mn_{0.7}Co_{0.2}Al_{0.1}O_2$ and $K_{0.45}MnO_2$.

**Samples**	**Mn average valence**
$K_{0.45}Mn_{0.7}Co_{0.2}Al_{0.1}O_2$	+ 3.786
$K_{0.45}MnO_2$	+ 3.55

Table S6. The voltage polarization calculation results of $K_{0.45}Mn_{0.7}Co_{0.2}Al_{0.1}O_2$ and $K_{0.45}MnO_2$.

	$K_{0.45}Mn_{0.7}Co_{0.2}Al_{0.1}O_2$				
Oxidation/Reduction peaks (V)	1.78/1.50	2.08/1.87	2.50/2.28	3.14/2.84	3.81/3.58
Voltage polarization	0.28	0.21	0.22	0.3	0.23
	$K_{0.45}MnO_2$				
Oxidation/Reduction peaks (V)	—	2.47/2.26	2.88/2.58	3.16/2.82	3.84/3.48
Voltage polarization	—	0.21	0.30	0.34	0.36

Table S7. Electrochemical performance of $K_{0.45}Mn_{0.7}Co_{0.2}Al_{0.1}O_2$ compared with other layered oxide cathodes in PIBs.

**Layered oxide cathode**	**Voltage range (V)**	**Discharge capacity (mAh g^{-1})/ current density (mA g^{-1})**	**Cycling performance capacity retention (%)/ cycles**	**Rate performance (mAh g^{-1})/ current density (mA g^{-1})**	**Ref**
**$K_{0.45}Mn_{0.7}Co_{0.2}Al_{0.1}O_2$**	**1.5-3.9**	**111/50**	**71.6/500**	**67/2000**	**This work**
P2-$K_{0.6}CoO_2$	1.5-4.0	87.2/20	86.9/1000	30.6/400	[6]
$K_{0.5}Mg_{0.15}[Mn_{0.8}Mg_{0.05}]O_2$	1.5-3.9	102/10	48.2/400	50.3/500	[7]
$K_{5/9}Mn_{7/9}Ti_{2/9}O_2$	1.5-4.2	178/20	73/100	52.2/500	[8]
P2-type $K_{0.75}Ni_{1/3}Mn_{2/3}O_2$	1.5-4.3	110/20	86/300	91/1400	[9]
$K_{0.6}Mn_{0.8}Ni_{0.1}Ti_{0.1}O_2$	1.5-4.2	118/10	88/100	48/500	[10]
P3-type $K_{0.5}Mn_{0.6}Co_{0.2}Fe_{0.1}Mg_{0.1}O_2$	1.5-3.9	106.1/50	91/150	57.8/2000	[11]

Table S8. The obtained resistance values for $K_{0.45}MnO_2$ and $K_{0.45}Mn_{0.7}Co_{0.2}Al_{0.1}O_2$ through fitting the EIS spectra along the equivalent circuit.

Samples	R_S(Ω)	R_{CT}(Ω)
$K_{0.45}Mn_{0.7}Co_{0.2}Al_{0.1}O_2$	37.4	1289
$K_{0.45}MnO_2$	58.6	2043

References

1. Kresse, G.; Furthmüller, J., Efficiency of ab-initio total energy calculations for metals and semiconductors using a plane-wave basis set. *Comput. Mater. Sci.* **1996**, *6*, 15-50.
2. Kresse, G.; Joubert, D., From ultrasoft pseudopotentials to the projector augmented-wave method. *Phys. Rev. B* **1999**, *59*, 1758-1775.
3. Perdew, J. P.; Burke, K.; Ernzerhof, M., Generalized Gradient Approximation Made Simple. *Phys. Rev. Lett.* **1996**, *77*, 3865-3868.
4. Monkhorst, H. J.; Pack, J. D., Special points for Brillouin-zone integrations. *Phys. Rev. B* **1976**, *13*, 5188-5192.
5. Henkelman, G.; Uberuaga, B. P.; Jónsson, H., A climbing image nudged elastic band method for finding saddle points and minimum energy paths. *J. Chem. Phys.* **2000**, *113*, 9901-9904.
6. Zhang, Z.; Hu, Q.; Liao, J.; Xu, Y.; Duan, L.; Tian, R.; Du, Y.; Shen, J.; Zhou, X., Uniform P2-$K_{0.6}CoO_2$ Microcubes as a High-Energy Cathode Material for Potassium-Ion Batteries. *Nano Lett.* **2023**, *23*, 694-700.
7. Luo, R.-J.; Li, X.-L.; Ding, J.-Y.; Bao, J.; Ma, C.; Du, C.-Y.; Cai, X.-Y.; Wu, X.-J.; Zhou, Y.-N., Suppressing Jahn-Teller distortion and phase transition of $K_{0.5}MnO_2$ by K-site Mg substitution for potassium-ion batteries. *Energy Storage Mater.* **2022**, *47*, 408-414.
8. Xu, Y.-S.; Zhang, Q.-H.; Wang, D.; Gao, J.-C.; Tao, X.-S.; Liu, Y.; Sun, Y.-G.; Gu, L.; Chang, B.-B.; Liu, C.-T.; Shi, S.-Q.; Cao, A.-M., Enabling reversible phase transition on $K_{5/9}Mn_{7/9}Ti_{2/9}O_2$ for high-performance potassium-ion batteries cathodes. *Energy Storage Mater.* **2020**, *31*, 20-26.
9. Jo, J. H.; Choi, J. U.; Park, Y. J.; Jung, Y. H.; Ahn, D.; Jeon, T.-Y.; Kim, H.; Kim, J.; Myung, S.-T., P2-$K_{0.75}[Ni_{1/3}Mn_{2/3}]O_2$ Cathode Material for High Power and Long Life Potassium-Ion Batteries. *Adv. Energy Mater.* **2020**, *10*, 1903605.
10. Xu, Y.-S.; Zhou, Y.-N.; Zhang, Q.-H.; Qi, M.-Y.; Guo, S.-J.; Luo, J.-M.; Sun, Y.-G.; Gu, L.; Cao, A.-M.; Wan, L.-J., Layered oxides with solid-solution reaction for high voltage potassium-ion batteries cathode. *Chem. Eng. J.* **2021**, *412*, 128735.
11. Xiao, Z.; Xia, F.; Xu, L.; Wang, X.; Meng, J.; Wang, H.; Zhang, X.; Geng, L.; Wu, J.; Mai, L., Suppressing the Jahn–Teller Effect in Mn-Based Layered Oxide Cathode toward Long-Life Potassium-Ion Batteries. *Adv. Funct. Mater.* **2022**, *32*, 2108244.
